# Machine Learning Prediction of Iron Deficiency Anemia in Chinese Premenopausal Women 12 Months after Sleeve Gastrectomy

**DOI:** 10.3390/nu15153385

**Published:** 2023-07-30

**Authors:** Yunhui Pan, Ronghui Du, Xiaodong Han, Wei Zhu, Danfeng Peng, Yinfang Tu, Junfeng Han, Yuqian Bao, Haoyong Yu

**Affiliations:** 1 Department of Endocrinology and Metabolism, Shanghai Sixth People’s Hospital Affiliated to Shanghai Jiao Tong University School of Medicine, Shanghai Diabetes Institute, Shanghai Clinical Center of Diabetes, Shanghai Key Laboratory of Diabetes Mellitus, Shanghai Key Clinical Center for Metabolic Disease, Shanghai 200233, China; 2 Department of General Surgery, Shanghai Sixth People’s Hospital Affiliated to Shanghai Jiao Tong University School of Medicine, Shanghai 200233, China; 3 Department of Endocrinology and Metabolism, Haikou Orthopedic and Diabetes Hospital of Shanghai Sixth People’s Hospital, Haikou 570300, China; 4 Department of Endocrinology and Metabolism, Tongji Hospital, School of Medicine, Tongji University, Shanghai 200065, China

**Keywords:** machine learning, support vector machine, iron deficiency anemia, sleeve gastrectomy

## Abstract

Premenopausal women, who account for more than half of patients for bariatric surgery, are at higher risk of developing postoperative iron deficiency anemia (IDA) than postmenopausal women and men. We aimed at establishing a machine learning model to evaluate the risk of newly onset IDA in premenopausal women 12 months after sleeve gastrectomy (SG). Premenopausal women with complete clinical records and undergoing SG were enrolled in this retrospective study. Newly onset IDA after surgery, the main outcome, was defined according to the age- and gender-specific World Health Organization criteria. A linear support vector machine model was developed to predict the risk of IDA after SG with the top five important features identified during feature selection. Four hundred and seven subjects aged 31.0 (Interquartile range (IQR): 26.0–36.0) years with a median follow-up period of 12 (IQR 7–13) months were analyzed. They were divided into a training set and a validation set with 285 and 122 individuals, respectively. Preoperative ferritin, age, hemoglobin, creatinine, and fasting C-peptide were included. The model showed moderate discrimination in both sets (area under curve 0.858 and 0.799, respectively, *p* < 0.001). The calibration curve indicated acceptable consistency between observed and predicted results in both sets. Moreover, decision curve analysis showed substantial clinical benefits of the model in both sets. Our machine learning model could accurately predict newly onset IDA in Chinese premenopausal women with obesity 12 months after SG. External validation was required before the model was used in clinical practice.

## 1. Introduction

During the last several decades, obesity has reached epidemic proportions in both developing and developed countries [1]. Although having a healthy lifestyle seems to be an ideal option to lose weight, bariatric surgery (BS) results in greater and sustained improvements in weight loss, obesity-associated complications, all-cause mortality, and quality of life compared with non-surgical treatment options [2]. BS involves different techniques leading to different effects on energy metabolism. Currently, sleeve gastrectomy (SG) is one of the most performed techniques in clinic.

Iron deficiency (ID) and iron deficiency anemia (IDA) have been observed at higher rates in patients with obesity compared with the general population. SG has been shown to exacerbate IDA with a prevalence as high as 18% [3] through a variety of mechanisms, including malabsorption due to gastric volume reduction and decreased gastric acid secretion [4], as well as impaired dietary tolerance of red meat [5]. Premenopausal women are at higher risk of developing ID and subsequently IDA due to menstrual blood losses after BS [6]. In our previous study, it has been reported that premenopausal women develop IDA more often after RYGB compared with postmenopausal women and men [7]. In addition, according to the data from the International Federation for the Surgery of Obesity (IFSO) global registry during 2015–2018, 77.1% of all patients who underwent BS were women [8]. In general, 70% of these female patients were premenopausal women. Therefore, premenopausal women accounted for more than half of the patients for BS. Furthermore, Knight et al. found IDA after BS was associated with more likelihood of hospitalization, higher risk of BS complications, and greater healthcare costs [9].

Due to the high incidence of IDA in premenopausal women after BS and the burden resulting from postoperative IDA, there is a growing need for tools to predict IDA after SG in premenopausal female patients. These tools could improve clinical decisions for necessary postoperative nutrition interventions. However, to our knowledge, there have been no predictive models available in clinical practice. In light of recent advances in machine learning (ML), predictive models developed from ML algorithms have been feasible for evaluating the prognosis of various diseases including stroke and myocardial infarction [10,11,12]. Based on the ML algorithm support vector machine (SVM), the advanced-DiaRem score for the prediction of diabetes remission after BS was developed by Aron-Wisnewsky et al. and had improved performance [13]. Thus, ML algorithms have been proposed as an alternative to developing predictive models.

The objective of this study was to establish a predictive model for newly onset IDA using machine learning based on the baseline clinicopathologic data of premenopausal female patients with obesity who underwent SG.

## 2. Materials and Methods

### 2.1. Study Design and Participants

A retrospective study was conducted on premenopausal women with obesity who underwent SG between 2015 and 2021 in a referral center. The inclusion criteria included the following: body mass index (BMI) ≥ 27.5 kg/m^2^; aged 18 to 50 years; complete preoperative and 1-year follow-up information. The exclusion criteria were as follows: vegetarian; underwent other bariatric surgeries; anemia at baseline; renal failure at baseline; incomplete preoperative information or lost to follow-up; premenopausal women with heavy menstrual bleeding after surgery (heavy menstrual bleeding was defined as a total blood loss per menstrual cycle that regularly exceeds 80 mL [14]); postoperative bleeding occurring within 30 days after surgery (it was defined as either a drop in hemoglobin levels (>30 g/L) and/or blood loss confirmed on intervention that required treatment [15]). Medical history, age, height, weight, BMI, blood pressure (BP), and current medications were recorded at baseline and after surgery. Glucose, C-peptide, glycated hemoglobin (HbA1c) levels, and lipid profiles were measured preoperatively and at 1 year postoperatively. Blood samples were collected in fasting state. Blood routine test was conducted by a fully automated hematology analyzer XN-350 (Sysmex, Kobe, Japan). White blood cell (WBC) count was measured by the flow cytometry method. Hemoglobin (Hb) was measured by cyanide-free sodium lauryl sulfate method. Plasma glucose concentration was measured by the glucose oxidase method. Serum insulin and C-peptide levels were quantified using radio-immunoassays. HbA1c level was measured by high-performance liquid chromatography with a VARIANT II Hemoglobin A1c analyzer (Bio-Rad Laboratories, Hercules, CA, USA). The levels of alanine aminotransferase (ALT), aspartate aminotransferase (AST), blood urea nitrogen (BUN), creatinine (Cr), blood uric acid (BUA), triglycerides (TG), total cholesterol, high-density lipoprotein cholesterol (HDL-c) and low-density lipoprotein cholesterol (LDL-c) were determined by applying standard enzymatic methods using a biochemical analyzer (7600-120; Hitachi, Tokyo, Japan). Serum levels of ferritin and iron were measured by electrochemiluminescence immunoassay using Modular E170 analyzer (Roche Diagnostics, Basel, Switzerland). Serum vitamin B12 and folic acid levels were performed with radioimmunoassay method (MP Biomedicals, Irvine, CA, USA). 

After operation, all patients were supplemented orally with 2 daily multivitamins/minerals (containing vitamin A 650 µg, vitamin B_2_ 1.4 mg, vitamin B_6_ 1.4 mg, vitamin B_12_ 3 µg, vitamin C 120 mg, vitamin E 18 mg, vitamin D 6 µg, folic acid 500 µg, magnesium 250 mg, zinc 14 mg, iron 18 mg), alfacalcidol (0.5 µg) and calcium carbonate with vitamin D (600 mg).

Type 2 diabetes (T2DM) was diagnosed according to the 1999 World Health Organization criteria: fasting plasma glucose (FPG) ≥ 7.0 mmol/L and/or 2 h plasma glucose (2hPG) ≥ 11.1 mmol/L [16]. Hypertension was defined as systolic BP ≥ 140 mmHg and/or diastolic BP ≥ 90 mmHg, or the use of antihypertensive medications.

Patients meeting inclusion criteria were randomly divided into training and validation sets with a ratio of 7:3 using the R function “createDataPartition” in the “caret” R package. The training set was used to establish the ML predictive model and the validation set was used to evaluate the performance of the model.

The Ethics Committee of our institution approved the study in accordance with the guidelines of the Declaration of Helsinki (World Medical Association). Informed consent was obtained from all participants included in the study.

### 2.2. Surgical Techniques

The surgical technique used in this study was laparoscopic SG, as described in a previous study [17]. All the surgeries were performed by the same surgical group in the referral center with the patients in the supine position. The gastric tube was created over a 37-Fr bougie using green and blue staples. Gastric section started 5 cm away from the pylorus towards the angle of His. Afterwards, the staple line was reinforced with running an absorbable suture.

### 2.3. Definitions of Anemia and Iron Deficiency Anemia

Anemia was defined by the age- and gender-specific World Health Organization (WHO) criteria, Hb < 12 g/dL in females [18]. IDA was defined as mean corpuscular volume (MCV) < 80 fl, mean corpuscular hemoglobin concentration (MCHC) < 27 g/dL, and ferritin < 30 ng/mL [19].

### 2.4. Data Pre-Processing and Feature Selection

The imbalance between two outcomes in the training set was mitigated by synthetic minority oversampling technique (SMOTE), which could create synthetic minority class samples. SMOTE was conducted by deploying the “SMOTE” function in the R package “DmWR”. Subsequently, baseline clinicopathologic data were used to build a random forest model and the importance of the features was ranked on the basis of mean decrease in GINI index. The top 5 features were included in the model learning.

### 2.5. Statistical Analysis

The sample size was estimated using the R package “pmsamplesize” for predictive model sample size calculation. Clinical characteristics are presented as mean ± standard deviation (SD) and median + interquartile range (IQR) for normally and non-normally distributed continuous variables, respectively; binominal variables are presented as frequencies and percentages. Shapiro–Wilk normality tests and histograms were used to verify whether the continuous variables had a normal distribution. Independent *t*-tests, chi-square tests, and Mann–Whitney U tests were performed to compare baseline characteristics between training and validation sets as well as between normal and newly onset IDA groups in the training set. Paired *t*-tests, Wilcoxon tests, and McNemar tests were performed to compare baseline and 1-year characteristics within normal and newly onset IDA groups in the training set.

For model learning, we used the linear SVM, which is a classification algorithm with acceptable accuracy under low computational power and small sample size. In order to detect overfitting and make alterations, if necessary, we performed tenfold cross-validation. Importance of variables included in the model was calculated by the varImp function of the R package “caret”. The discriminative ability of the model was evaluated by the area under curve (AUC) derived from receiver operating characteristic (ROC) curve. The significance of the AUCs compared to 0.5 was tested by the DeLong method and *p* values were generated. Calibration was validated by performing calibration curve analysis with bootstrapping to assess the agreement between model predictive and actual probability. Furthermore, decision curve analysis (DCA) was performed to evaluate the net benefits of the model. Finally, we developed an application based on our model by using the R package “shiny”. All statistical analysis was performed using IBM SPSS Statistics 25.0 (IBM Corp., Armonk, NY, USA) and R statistical software 4.1.2 (R Foundation for Statistical Computing, Vienna, Austria). The ML predictive model was constructed using the “caret” package, and DCA was conducted using the “rmda” package. *p* value < 0.05 (two-sided) was considered statistically significant.

## 3. Results

### 3.1. Clinical Characteristics of Study Subjects

**Figure 1:**
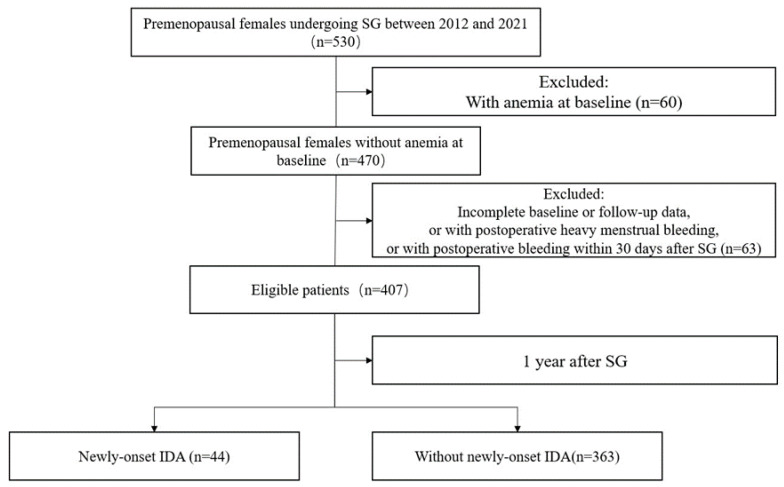
Flowchart of study participants.

In accordance with aforementioned inclusion and exclusion criteria, 407 eligible patients were actually seen both at baseline and last follow-up in the analysis (Figure 1). At baseline, median age was 31.0 (IQR: 26.0–36.0) years. Median BMI was 37.0 (IQR 33.3–41.3) kg/m^2^. Median Hb at baseline was 136.0 (IQR: 130.0–142.0) g/L. The prevalence of T2DM was 35.9%. Median follow-up was 12 (IQR 7–13) months (Table 1). Of these patients, forty-four individuals (10.8%) had newly onset IDA and 363 (89.2%) did not.

All eligible patients were divided into a training set and a validation set including 285 individuals and 122 individuals, respectively. According to the calculation executed by the package “pmsamplesize”, the minimum sample size was 199 for the training set to build a predictive model including 5 parameters when R^2^ of the model was set to be 0.2. The sample size of the training set was able to meet the minimum requirement.

The baseline clinical characteristics of both sets are shown in Table 1. There existed no significant differences in anthropometric and biochemical parameters, the prevalence of T2DM, hypertension, and usage of related agents between the two datasets. In the training set, 26 individuals (9.1%) had newly onset IDA and 259 (90.9%) did not. Patients who exhibited newly onset IDA had significantly lower preoperative systolic BP (*p* = 0.032), white blood cell count (*p* = 0.048), hemoglobin (*p* = 0.021), ferritin (*p* < 0.001), Cr (*p* = 0.006), BUA (*p* = 0.005), HbA1c (*p* = 0.016), fasting insulin (*p* = 0.013) and fasting C-peptide (FCP) (*p* = 0.009) (Table 2).

### 3.2. The SVM Model Construction and Evaluation

**Figure 2:**
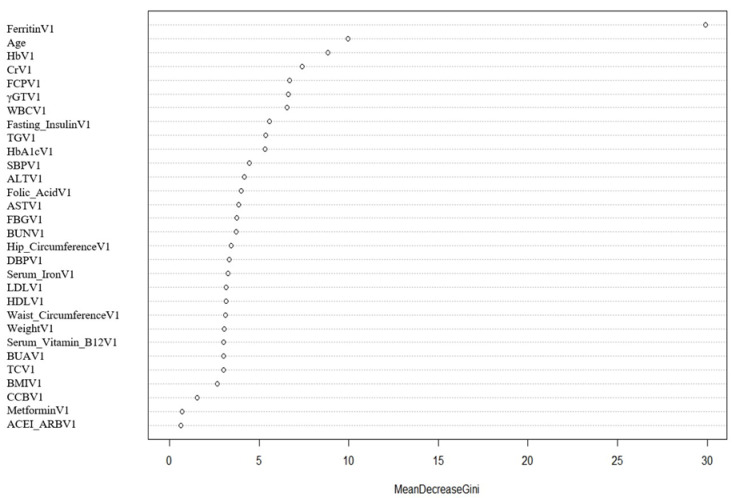
Feature importance during the process of feature selection. BMI = body mass index; SBP = systolic blood pressure; DBP = diastolic blood pressure; Hb = hemoglobin; WBC = white blood cell; ALT = alanine transaminase; AST = aspartate transaminase; γ-GT = γ-glutamyl transpeptidase; BUN = blood urea nitrogen; Cr = creatinine; BUA = blood uric acid; FPG = fasting plasma glucose; FCP = fasting C-peptide; HbA1c = glycated hemoglobin A1c; TG = total triglycerides; TC = total cholesterol; HDL-c = high-density lipoprotein; LDL-c = low-density lipoprotein; ACEI = angiotensin-converting enzyme inhibitors; ARB = angiotensin II receptor blockers; CCB = calcium channel blockers.

**Figure 3:**
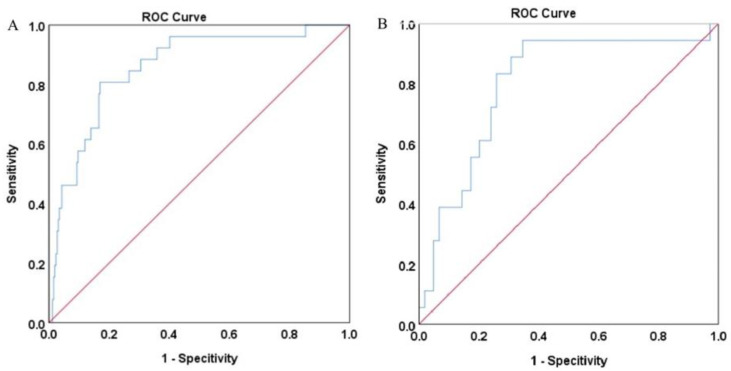
Receiver operating characteristic curve for the prediction model in both datasets. (**A**) Training set. (**B**) Validation set. Area under the curve was 0.858 (95% confidence interval 0.784–0.931) in the training set. Area under the curve was 0.799 (95% confidence interval 0.689–0.910) in the validation set.

**Figure 4:**
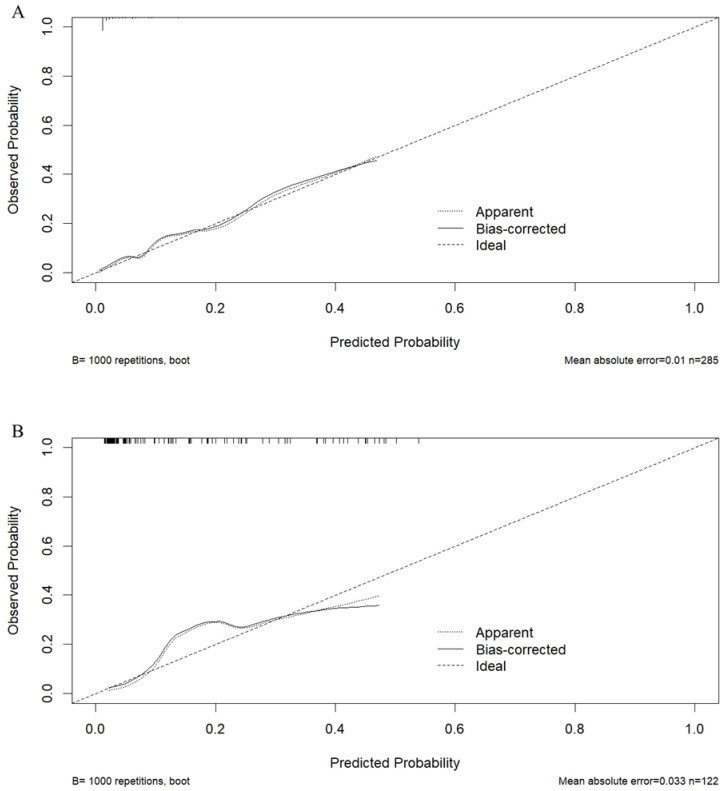
Calibration of the model for newly onset IDA after SG. (**A**) Training set. (**B**) Validation set. The x-axis shows the predicted probability of newly onset IDA after SG, and the y-axis shows the observed probability of newly onset IDA after SG.

**Figure 5:**
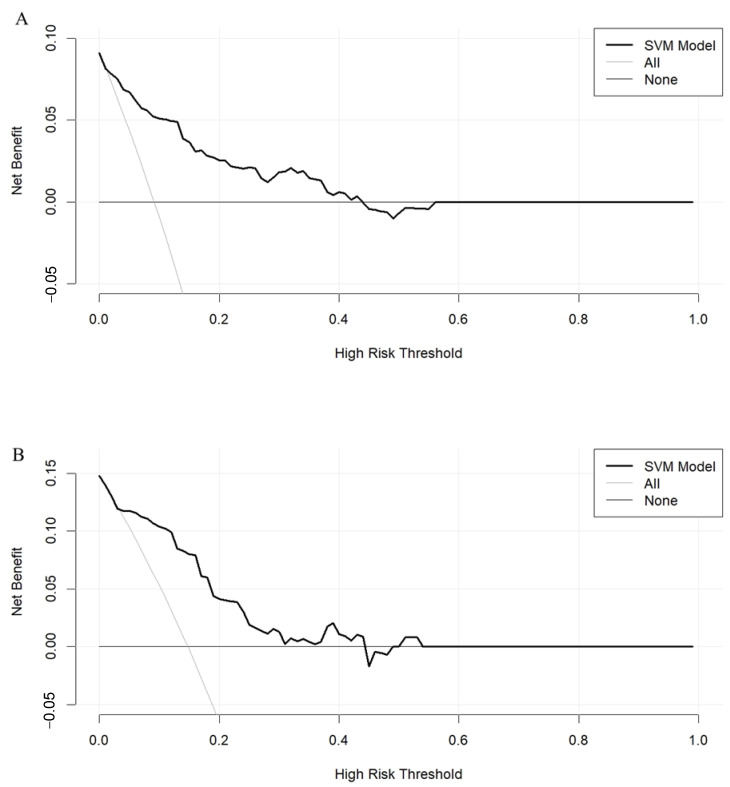
Decision curve analysis of the model for newly onset IDA after SG. (**A**) Training set. (**B**) Validation set.

**Figure 6:**
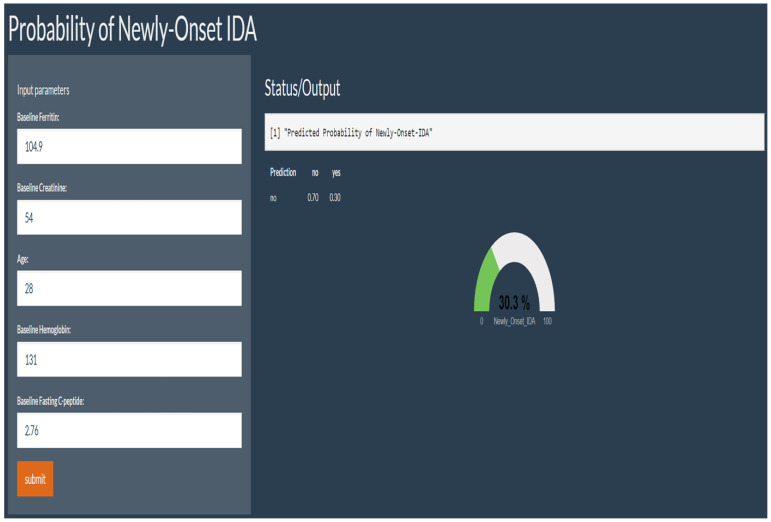
The application based on the machine learning model. After parameters were input into the panel on the left, the application could calculate the probability of postoperative newly onset IDA.

After SMOTE of the training set by setting the parameter “perc.over” as 500 and the parameter “perc.under” as 120, a dataset consisting of 50%, 156 samples with a normal outcome and 50%, 156 samples with newly onset IDA after SG was created. The feature importance for predicting IDA using a random forest algorithm after SMOTE was shown in Figure 2. In the feature selection step, preoperative ferritin, age, hemoglobin, Cr, and FCP were the top five features, which were then included in the SVM model. Their importance was shown in Table 3. Preoperative ferritin was the most important contributor to the model. In the training set, the AUC was 0.858 (95% CI 0.784–0.931, *p* < 0.001; Figure 3A). The calibration curve of the model in the training set was close to the ideal diagonal line and the mean absolute error was 0.01, which was close to 0 (Figure 4A). These indicated acceptable consistency between observed and model-predicted results in the training set, and the model was well calibrated. DCA curve indicated that the model added net benefits compared with the treat-all-patients scheme and the treat-none scheme (Figure 5A). The importance of these features in the SVM model was calculated and preoperative ferritin was the most important among them. In the validation set, the AUC was 0.799 (95% CI 0.689–0.910, *p* < 0.001; Figure 3B). The calibration curve of the model in the validation set was also close to the ideal diagonal line and the mean absolute error was 0.03, which was also close to 0 (Figure 4B). These indicated fair consistency between observed and model-predicted results in the validation set, and the model was well calibrated. Moreover, the DCA curve showed net benefits of the predictive model in the validation set as well (Figure 5B). Finally, an application based on our model was developed by using the “shiny” package. The application could calculate the probability of postoperative newly onset IDA after parameters were input into the panel on the left (Figure 6).

## 4. Discussion

To the best of our knowledge, the study is the first to establish an ML model for accurately predicting newly onset IDA after SG in premenopausal female patients with obesity. IDA is a common nutritional problem and complication after BS, especially in premenopausal females [7]. Gowanlock et al. suggested IDA was reported in 16% of patients after BS in their cohort with 388 subjects [20]. With regard to different surgical procedures, Kwon et al. showed there were no significant differences in the risk of postoperative anemia or ID between gastric bypass and SG [21]. According to Nie et al., the pooled prevalence of anemia increased to 12% at 12 months after SG, and ferritin deficiency was strongly correlated with anemia [22]. In our cohort, the SG premenopausal patients had a reported IDA incidence of 10.8% post-operatively, which is similar to previous studies.

As for sample size calculation of the training set, in the function “pmsamplesize”, the parameter “prevalence” was set as 0.11, which was approximately the prevalence of newly onset IDA in our cohort, and the parameter “parameters” was set as 5 in accordance with the expected number of features in our model. According to the calculation of the function, the maximum R^2^ for an outcome proportion of 0.11 was 0.5. As was suggested by Riley et al. covering the sample size calculation for developing a clinical prediction model in detail [23], the anticipated R^2^ of the model could be set as 50% of the maximum R^2^ when the training dataset included direct measures of the clinical process involved. In this study, direct measures of IDA such as ferritin and Hb were included in the training set. Thus, the anticipated R^2^ could be set as 0.25, or 50% of 0.5. With this, the anticipated R^2^ and aforementioned parameters, the minimum sample size of the training set was 172, smaller than the actual size. Actually, the anticipated R^2^ here was set as 0.2, or 40% of 0.5, which was more conservative than the suggestion in the literature. Moreover, when the anticipated R^2^ was set as a conservative one, 0.15, or 30% of 0.5, the minimum sample size required for the training set was 275, again smaller than the actual one. In summary, the sample size of our training set was adequate for model construction.

When the model was developed, we considered the computation complexity and ease of prediction. Additionally, considering the relatively short consulting time for each patient in the outpatient of our center and the short hospital stay, a simpler model might be more appropriate to assist the clinicians or the team for BS in evaluating the risk of newly onset IDA than a complex one. Apart from this, because of the limited sample size, our training set might not be capable of supporting the construction of a complex model. For instance, according to the calculation by the “pmsamplesize” package, the minimal sample size of the training set for building a 10-feature model was over 300, larger than the actual size. Based on these considerations and conditions, we performed feature selection with the help of the Gini index decrease calculated by random forest.

The present study suggests that preoperative serum ferritin, Hb, age, FCP, and Cr levels relate to newly onset IDA in premenopausal patients with obesity. Firstly, in clinics, ferritin is predominantly utilized as a serum marker of total body iron stores. In cases of iron deficiency and overload, serum ferritin serves a critical role in both diagnosis and management. In a retrospective study involving 2116 subjects who underwent gastric bypass, McCracken et al. found preoperative low ferritin (defined as <13 ng/mL for females and <30 ng/mL for males) was a significant factor associated with postoperative severe anemia in both univariate and multivariate analysis [24]. Thus, preoperative low ferritin was included in the scoring algorithm developed by them for the prediction of postoperative severe anemia. In another study that set out to determine the factors associated with IDA after BS including SG, gastric bypass, and duodenal switch, Gowanlock et al. showed low baseline ferritin level was associated with an increased risk of IDA with a mean follow-up of 31 months after BS. A baseline ferritin level of less than 30 mg/L was associated with a higher risk of IDA, whereas a ferritin level of 156 mg/L or greater carried a minimal risk of IDA even after 6 years of follow-up [20]. In our study with a median follow-up of 12 months, the newly onset IDA group in the training set also had significantly lower serum ferritin levels at baseline compared with the normal group, even though median ferritin levels were in the normal range in both groups.

Secondly, preoperative Hb per se can predict postoperative anemia or IDA. Lee et al. investigated the factors affecting anemia development after BS including gastric bypass, gastric binding, and SG in their retrospective cohort with 442 subjects, they found pre-operative optimal value of Hb 156 g/L was able to predict future anemia in patients with morbid obesity 2 years after BS [25]. In an aforementioned study by Gowanlock et al., they reported lower preoperative Hb (Hb < 12 g/dL in females) was correlated with an increased risk of postoperative IDA [20]. Moreover, a recent study by Ben-Porat et al. with 121 subjects showed a lower pre-operative Hb level was an independent factor associated with anemia during pregnancy 2 years after SG [26]. Thus, the inclusion of preoperative Hb as one of the predictors in our model corroborated these previous findings about the association between preoperative Hb and anemia or IDA after BS. In addition, the median Hb in our newly onset IDA group of the training set at baseline was 131.0 g/L, which was on par with the pre-operative Hb level of patients developing postoperative anemia in the SG cohort present in the study by Ben-Porat et al. Therefore, it might be necessary to prevent the development of IDA in a premenopausal patient with an Hb level of around 130 g/L before SG.

Age is another factor included in our predictive model. The association between age and anemia or IDA after BS has been investigated in previous studies. In a large cohort study, aimed at exploring possible factors correlating to the risk of anemia after BS including gastric bypass, SG, and gastric banding, Bailly et al. identified younger age (defined as <52) as a factor for the occurrence of anemia after BS in their cohort with 306,298 patients [27]. In an East Asia cohort with 4373 subjects, Wang et al. found the incidence of post-BS anemia increased among patients in young-aged (defined as 20–29 years) and middle-aged (defined as 30–64 years) groups [28]. The aforementioned study by Gowanlock et al., which focused on the predictors of IDA after BS in a cohort with a mean age of 46, also reported young age was associated with an increased risk of IDA [20]. In our study, the median age of the newly onset IDA group in the training set was 33.5. It was a rather young age in comparison to previous studies and was located in the previously reported age groups correlating to the risk of anemia or IDA after BS. Therefore, it might be necessary for healthcare providers to take measures for the prevention of IDA in young female patients after SG.

Preoperative FCP and Cr were the other two factors included in our model. As for FCP, relatively lower FCP may indicate impaired pancreatic beta-cell function. In a cross-sectional study by Chung et al. with 1300 participants, lower FCP was reported to be associated with more severe anemia in type 2 diabetes patients [29]. Dysregulated iron metabolism, which could be caused by insulin resistance in patients with obesity or metabolic syndrome through various mechanisms [30], might further deteriorate when beta-cell function declined. Therefore, preoperative FCP may be a factor associated with IDA in premenopausal patients after SG. With regard to preoperative Cr, relatively lower serum Cr may reflect decreased skeletal muscle proportion or lower red meat intake as Cr is a measure of protein metabolism in subjects with normal renal function. A recently published study by Ikeda-Taniguchi et al. found malnourished patients with skeletal muscle loss showed functional iron deficiency such as iron binding and utilization capacity intolerance [31]. Thus, preoperative Cr may be a predictor of IDA after SG in premenopausal patients.

With regard to the net benefit of the model, the DCA curves showed that compared to treating all patients empirically (the grey line), treating patients after the prediction of the SVM model could produce more benefits in both sets (the black line). Moreover, the intervention or prevention of IDA mainly involved nutritional arrangements and iron supplementations, which were effective and did not have many costs, inconveniences, or many adverse effects. Therefore, the probability or risk threshold of taking these measures might be low, possibly <0.2, meaning the patients might opt for intervention in this range of newly onset IDA probability. Within this range, treating patients after the prediction of the model performed better than treating all patients empirically, which might indicate the clinicians could persuade patients from overconcern about postoperative IDA and keep them from postoperative overtreatment for IDA prevention with the assistance of the model.

This study had a couple of limitations. First, this was a single-center retrospective study, thus potentially introducing selection bias. The generalization of the results to the entire bariatric population required external validation in multi-center cohorts with a larger sample size. Second, the follow-up period of our study was relatively short. Hence, the predictive capacity of our model in long-term IDA risk after SG called for further studies. Third, preoperative dietary information with a quantitative questionnaire was not collected. Due to this, the association between preoperative dietary structure and postoperative IDA risk could not be evaluated. Fourth, we did not make a body composition assessment, especially skeletal muscle, by magnetic resonance or dual-energy X-ray methods. Therefore, whether there was preoperative skeletal muscle loss in our cohort was not clear.

This study also had strengths. First, this study focused exclusively on premenopausal women, due to this population having the highest postoperative IDA incidence. Second, this study only included only patients who underwent SG, which is the most commonly recommended surgery for obesity according to the guidelines. Hence, we avoided the bias seen in other studies that evaluated the whole population with multiple types of BS, which are associated with varying metabolic effects on IDA. Third, instead of merely listing the risk factors associated with postoperative IDA, these factors were developed into a predictive model and a feasible tool for clinical practice in our study. Our ML model precisely predicted the probability of IDA at about 1 year after SG. Healthcare providers could in advance discuss the necessary postoperative arrangements with patients at risk of IDA after SG and essential healthcare resources could be assigned to these patients under the direction of our model. The multidisciplinary team for BS could plan for postoperative nutritional management and iron supplementation arrangements for the early postoperative intervention of patients at risk of IDA after SG. Furthermore, the surgery team could also take measures to mitigate the previously predicted postoperative IDA risk of the patients and then conduct a re-evaluation by using the model again. Therefore, the team could ensure that the patients would undergo the surgery at a lower risk of postoperative IDA.

In conclusion, we first devised an ML predictive model which consisted of preoperative ferritin, age, hemoglobin, Cr, and FCP and resulted in accurate prediction of IDA in premenopausal female patients with obesity after SG and may provide a reference in terms of preventive interventions. Our model had acceptable discrimination, calibration, and net benefits in predicting newly onset IDA after SG. With the pre-operative evaluation of the postoperative risk of newly onset IDA, the multidisciplinary team for BS could discuss postoperative nutritional management and iron supplementation arrangements beforehand for the early postoperative intervention of patients at risk of IDA after SG. Apart from this, the surgery team could also operate on the patients after taking measures to mitigate their predicted postoperative IDA risk. Further validation in other ethnic groups will be of interest.

## Figures and Tables

**Table 1:** Baseline clinical characteristics of all patients in the training set and validation set.

	All Patients (n = 407)	Training Set (n = 285)	Validation Set (n = 122)
Median follow-up (months)	12 (7, 13)	12 (7, 13)	12 (7, 13)
Newly onset IDA (N, %)	44 (10.8)	26 (9.1)	18 (14.8)
T2DM (N, %)	146 (35.9)	107 (37.5)	39 (32.0)
Hypertension (N, %)	96 (23.6)	69 (24.2)	27 (22.1)
Age (y)	31.0 (26.0, 36.0)	31.0 (26.5, 36.0)	31.0 (25.8, 37.0)
Weight (kg)	100.0 (89.0, 113.5)	100.0 (89.4, 113.0)	100.5 (88.9, 114.5)
BMI (kg/m^2^)	37.0 (33.3, 41.3)	36.7 (33.4, 41.5)	37.5 (33.3, 41.0)
Waist circumference (cm)	112.0 (103.0, 123.0)	112.0 (103.0, 123.0)	112.0 (103.4, 124.0)
Hip circumference (cm)	116.0 (108.0,124.0)	116.0 (108.0, 124.0)	117.0 (108.8, 123.0)
SBP (mmHg)	130.0 (120.0, 140.0)	129.0 (120.0, 140.0)	130.0 (120.0, 139.3)
DBP (mmHg)	84.0 (78.0, 92.0)	85.0 (78.0, 94.0)	84.0 (77.8, 90.0)
WBC (×10^9^/L)	7.9 (6.7, 9.3)	8.0 (7.0, 9.4)	7.7 (6.6, 9.1)
Hb (g/L)	136.0 (130.0, 142.0)	137.0 (130.5, 143.0)	135.0 (129.0, 140.3)
ALT (U/L)	34.0 (22.0, 63.0)	35.0 (22.0, 60.5)	33.5 (22.0, 64.3)
AST (U/L)	24.0 (17.0, 38.0)	23.0 (18.0, 41.0)	24.0 (16.8, 35.3)
γ-GT (U/L)	34.0 (23.0, 53.0)	35.0 (23.0, 58.0)	34.0 (23.0, 46.3)
BUN (mmol/L)	4.6 (3.9, 5.5)	4.6 (3.9, 5.5)	4.6 (3.8, 5.4)
Cr (μmol/L)	54.0 (47.9, 61.0)	54.0 (47.7, 61.0)	54.0 (48.0, 60.2)
BUA (μmol/L)	385.0 (329.0, 445.0)	389.0 (327.5, 450.5)	379.5 (333.8, 432.0)
TC (mmol/L)	5.1 (4.5, 5.9)	5.2 (4.5, 5.8)	5.1 (4.5, 5.9)
TG (mmol/L)	1.5 (1.1, 2.2)	1.5 (1.2, 2.2)	1.4 (1.1, 2.2)
HDL-c (mmol/L)	1.1 (0.9, 1.3)	1.1 (0.9, 1.3)	1.1 (0.9, 1.3)
LDL-c (mmol/L)	3.2 (2.7, 3.8)	3.2 (2.7, 3.8)	3.2 (2.7, 3.8)
FPG (mmol/L)	5.6 (5.0, 7.0)	5.6 (5.0, 7.2)	5.6 (5.0, 6.6)
HbA1c (%)	5.8 (5.4, 6.8)	5.9 (5.4, 6.9)	5.7 (5.4, 6.6)
Fasting insulin (mIU/L)	27.1 (18.8, 40.3)	26.5 (18.2, 41.2)	28.3 (20.4, 37.1)
Fasting C-peptide (ng/mL)	4.0 (3.2, 5.0)	3.9 (3.2, 5.0)	4.1 (3.2, 4.9)
Serum folic acid (μg/L)	7.2 (4.7, 10.4)	6.9 (4.7, 10.3)	7.8 (4.7, 10.9)
Serum vitamin B12 (ng/L)	530.9 (410.1, 669.8)	531.0 (409.0, 660.8)	527.8 (409.6, 683.4)
Serum iron (μmol/L)	14.4 (11.5, 18.8)	14.6 (11.7, 19.0)	14.1 (11.0, 18.5)
Ferritin (ng/mL)	106.8 (60.0, 175.4)	109.8 (60.5, 174.9)	103.7 (57.1, 176.2)
**Antidiabetic agents (N, %)**			
Sulfonylurea	16 (3.9)	12 (4.2)	4 (3.3)
Metformin	69 (17.0)	47 (16.5)	22 (18.0)
α-glucosidase inhibitors	18 (4.4)	14 (4.9)	4 (3.3)
Thiazolidinediones	7 (1.7)	4 (1.4)	3 (2.5)
DPP-IV inhibitors	5 (1.2)	4 (1.4)	1 (0.8)
SGLT-2 inhibitors	7 (1.7)	3 (1.1)	4 (3.3)
Insulin	28 (6.9)	20 (7.0)	8 (6.6)
GLP-1RAs	13 (3.2)	10 (3.5)	3 (2.5)
**Antihypertensive agents (N, %)**			
ACEI/ARB	34 (8.4)	23 (8.1)	11 (9.0)
β-blockers	12 (2.9)	8 (2.8)	4 (3.3)
CCB	34 (8.4)	22 (7.7)	12 (9.8)
Diuretics	4 (1.0)	2 (0.7)	2 (1.6)
**Lipid-lowering agents (N, %)**			
Statins	7 (1.7)	3 (1.1)	4 (3.3)
Fibrates	8 (2.0)	5 (1.8)	3 (2.5)

Characteristics are presented as mean ± standard deviation (SD) or median + interquartile range (IQR) for normally and non-normally distributed continuous variables. T2DM = type 2 diabetes mellitus; BMI = body mass index; BP = blood pressure; SBP = systolic blood pressure; DBP = diastolic blood pressure; WBC = white blood cell; Hb = hemoglobin; ALT = alanine transaminase; AST = aspartate transaminase; γ-GT = γ-glutamyl transpeptidase; BUN = blood urea nitrogen; Cr = creatinine; BUA = blood uric acid; FPG = fasting plasma glucose; TG = total triglycerides; TC = total cholesterol; HDL-c = high-density lipoprotein; LDL-c = low-density lipoprotein; HbA1c = glycated hemoglobin A1c; DPP-IV = dipeptidyl peptidase IV; SGLT-2 = sodium-glucose cotransporter-2; GLP-1RA = glucagon-like peptide 1 receptor agonists; ACEI = angiotensin-converting enzyme inhibitors; ARB = angiotensin II receptor blockers; CCB = calcium channel blockers.

**Table 2:** Clinical characteristics of normal and newly onset IDA groups in the training set at baseline.

	Normal (n = 259)	Newly Onset IDA (n = 26)	*p* between Groups
T2DM (N, %)	98 (37.8)	9 (34.6)	0.746
Hypertension (N, %)	60 (23.2)	9 (34.6)	0.194
Age (y)	31.0 (26.0, 36.0)	33.5 (29.5, 36.0)	0.191
Weight (kg)	101.5 (90.0, 113.0)	94.7 (87.1, 113.8)	0.279
BMI (kg/m^2^)	36.9 (33.3, 41.8)	35.9 (33.3, 40.2)	0.440
Waist circumference (cm)	113.0 (103.0, 123.0)	110.0 (101.8, 121.5)	0.549
Hip circumference (cm)	117.0 (108.0, 124.0)	112.0 (108.3, 121.5)	0.295
SBP (mmHg)	130.0 (120.0, 140.0)	122.5 (113.0, 132.8)	0.032
DBP (mmHg)	86.0 (78.0, 94.0)	80.0 (72.3, 90.5)	0.182
WBC (×10^9^/L)	8.1 (7.0, 9.5)	7.5 (6.0, 8.5)	0.048
Hb (g/L)	137.0 (131.0, 144.0)	131.0 (125.0,142.0)	0.021
ALT (U/L)	35.0 (23.0, 61.0)	32.0 (17.5, 63.8)	0.513
AST (U/L)	24.0 (18.0, 41.0)	22.0 (17.5, 46.8)	0.636
γ-GT (U/L)	35.0 (24.0, 59.0)	27.0 (21.0, 53.3)	0.075
BUN (mmol/L)	4.7 (4.0, 5.5)	4.3 (3.6, 5.3)	0.101
Cr (μmol/L)	54.5 (48.0, 62.0)	49.5 (46.0, 55.2)	0.006
BUA (μmol/L)	391.0 (331.0, 455.0)	326.0 (303.0, 412.5)	0.005
TC (mmol/L)	5.2 (4.5, 5.8)	5.1 (4.5, 6.4)	0.940
TG (mmol/L)	1.5 (1.1, 2.2)	1.7 (1.2, 2.5)	0.471
HDL-c (mmol/L)	1.1 (0.9, 1.3)	1.0 (0.9, 1.4)	0.975
LDL-c (mmol/L)	3.2 (2.7, 3.8)	3.3 (2.8, 3.7)	0.755
FPG (mmol/L)	5.6 (5.0, 7.3)	5.4 (4.9, 7.2)	0.712
HbA1c (%)	6.0 (5.4, 7.1)	5.5 (5.3, 6.3)	0.016
Fasting insulin (mIU/L)	27.5 (18.8, 42.0)	17.6 (13.8, 28.5)	0.013
Fasting C-peptide (ng/mL)	4.0 (3.3, 5.1)	3.2 (2.4, 4.2)	0.009
Serum folic acid (μg/L)	6.8 (4.6, 10.3)	8.1 (5.2, 10.5)	0.540
Serum vitamin B12 (ng/L)	531.0 (410.1, 657.5)	551.0 (405.3, 740.3)	0.411
Serum iron (μmol/L)	14.8 (11.8, 18.9)	12.3 (8.2, 20.5)	0.104
Ferritin (ng/mL)	115.6 (71.3, 196.5)	41.9 (29.0, 67.1)	<0.001
**Antidiabetic agents (N, %)**			
Sulfonylurea	12 (4.6)	0 (0)	0.610
Metformin	44 (17.0)	3 (11.5)	0.662
α-glucosidase inhibitors	14 (5.4)	0 (0)	0.625
Thiazolidinediones	4 (1.5)	0 (0)	1.000
DPP-IV inhibitors	4 (1.5)	0 (0)	1.000
SGLT-2 inhibitors	3 (1.2)	0 (0)	1.000
Insulin	18 (6.9)	2 (7.7)	1.000
GLP-1RAs	9 (3.5)	1 (3.8)	1.000
**Antihypertensive agents (N, %)**			
ACEI/ARB	20 (7.7)	3 (11.5)	0.762
β-blockers	6 (2.3)	2 (7.7)	0.337
CCB	20 (7.7)	2 (7.7)	1.000
Diuretics	1 (0.4)	1 (3.8)	0.434
**Lipid-lowering agents (N, %)**			
Statins	2 (0.8)	1 (3.8)	0.648
Fibrates	5 (1.9)	0 (0)	1.000

Characteristics are presented as mean ± standard deviation (SD) or median + interquartile range (IQR) for normally and non-normally distributed continuous variables. T2DM = type 2 diabetes mellitus; BMI = body mass index; BP = blood pressure; SBP = systolic blood pressure; DBP = diastolic blood pressure; WBC = white blood cell; Hb = hemoglobin; ALT = alanine transaminase; AST = aspartate transaminase; γ-GT = γ-glutamyl transpeptidase; BUN = blood urea nitrogen; Cr = creatinine; BUA = blood uric acid; FPG = fasting plasma glucose; TG = total triglycerides; TC = total cholesterol; HDL-c = high-density lipoprotein; LDL-c = low-density lipoprotein; HbA1c = glycated hemoglobin A1c; DPP-IV = dipeptidyl peptidase IV; SGLT-2 = sodium-glucose cotransporter-2; GLP-1RA = glucagon-like peptide 1 receptor agonists; ACEI = angiotensin-converting enzyme inhibitors; ARB = angiotensin II receptor blockers; CCB = calcium channel blockers.

**Table 3:** Importance of variables in the model calculated by varImp function.

Variables	Importance
**Ferritin**	0.86
**Hb**	0.69
**Age**	0.68
**Fasting C-peptide**	0.64
**Cr**	0.52

Hb = hemoglobin; Cr = creatinine.

## Data Availability

Not applicable.

## References

[1] Ng M., Fleming T., Robinson M., Thomson B., Graetz N., Margono C., Mullany E.C., Biryukov S., Abbafati C., Abera S.F. (2014). Global, regional, and national prevalence of overweight and obesity in children and adults during 1980–2013: A systematic analysis for the Global Burden of Disease Study 2013. Lancet.

[2] Arterburn D.E., Telem D.A., Kushner R.F., Courcoulas A.P. (2020). Benefits and Risks of Bariatric Surgery in Adults: A Review. JAMA.

[3] Jáuregui-Lobera I. (2013). Iron deficiency and bariatric surgery. Nutrients.

[4] Obinwanne K.M., Fredrickson K.A., Mathiason M.A., Kallies K.J., Farnen J.P., Kothari S.N. (2014). Incidence, treatment, and outcomes of iron deficiency after laparoscopic Roux-en-Y gastric bypass: A 10-year analysis. J. Am. Coll. Surg..

[5] Decker G.A., Swain J.M., Crowell M.D., Scolapio J.S. (2007). Gastrointestinal and nutritional complications after bariatric surgery. Am. J. Gastroenterol..

[6] Levi M., Rosselli M., Simonetti M., Brignoli O., Cancian M., Masotti A., Pegoraro V., Cataldo N., Heiman F., Chelo M. (2016). Epidemiology of iron deficiency anaemia in four European countries: A population-based study in primary care. Eur. J. Haematol..

[7] Yu H., Du R., Zhang N., Zhang M., Tu Y., Zhang L., Bao Y., Han J., Zhang P., Jia W. (2016). Iron-Deficiency Anemia After Laparoscopic Roux-en-Y Gastric Bypass in Chinese Obese Patients with Type 2 Diabetes: A 2-Year Follow-Up Study. Obes. Surg..

[8] Welbourn R., Hollyman M., Kinsman R., Dixon J., Cohen R., Morton J., Ghaferi A., Higa K., Ottosson J., Pattou F. (2021). Bariatric-Metabolic Surgery Utilisation in Patients with and without Diabetes: Data from the IFSO Global Registry 2015–2018. Obes. Surg..

[9] Knight T., D’Sylva L., Moore B., Barish C.F. (2015). Burden of Iron Deficiency Anemia in a Bariatric Surgery Population in the United States. J. Manag. Care Spec. Pharm..

[10] Liu J., Chen X., Guo X., Xu R., Wang Y., Liu M. (2022). Machine learning prediction of symptomatic intracerebral hemorrhage after stroke thrombolysis: A cross-cultural validation in Caucasian and Han Chinese cohort. Ther. Adv. Neurol. Disord..

[11] Heo J., Yoon J.G., Park H., Kim Y.D., Nam H.S., Heo J.H. (2019). Machine Learning-Based Model for Prediction of Outcomes in Acute Stroke. Stroke.

[12] Khera R., Haimovich J., Hurley N.C., McNamara R., Spertus J.A., Desai N., Rumsfeld J.S., Masoudi F.A., Huang C., Normand S.L. (2021). Use of Machine Learning Models to Predict Death after Acute Myocardial Infarction. JAMA Cardiol..

[13] Aron-Wisnewsky J., Sokolovska N., Liu Y., Comaneshter D.S., Vinker S., Pecht T., Poitou C., Oppert J.M., Bouillot J.L., Genser L. (2017). The advanced-DiaRem score improves prediction of diabetes remission 1 year post-Roux-en-Y gastric bypass. Diabetologia.

[14] Sriprasert I., Pakrashi T., Kimble T., Archer D.F. (2017). Heavy menstrual bleeding diagnosis and medical management. Contracept. Reprod. Med..

[15] Straatman J., Verhaak T., Demirkiran A., Harlaar N.J., Cense H.A., Jonker F.H.W., Dutch Audit for Treatment of Obesity (DATO) Research Group (2022). Risk factors for postoperative bleeding in bariatric surgery. Surg. Obes. Relat. Dis..

[16] Alberti K.G., Zimmet P.Z. (1998). Definition, diagnosis and classification of diabetes mellitus and its complications. Part 1: Diagnosis and classification of diabetes mellitus provisional report of a WHO consultation. Diabet. Med..

[17] Di J., Wang C., Zhang P., Han X., Liu W., Zhang H. (2018). The middle-term result of laparoscopic sleeve gastrectomy in Chinese obesity patients in a single hospital, with the review of literatures and strategy for gastric stenosis. Ann. Transl. Med..

[18] Bermejo F., García-López S. (2009). A guide to diagnosis of iron deficiency and iron deficiency anemia in digestive diseases. World J. Gastroenterol..

[19] Weiss G., Goodnough L.T. (2005). Anemia of chronic disease. N. Engl. J. Med..

[20] Gowanlock Z., Lezhanska A., Conroy M., Crowther M., Tiboni M., Mbuagbaw L., Siegal D.M. (2020). Iron deficiency following bariatric surgery: A retrospective cohort study. Blood Adv..

[21] Kwon Y., Ha J., Lee Y.H., Kim D., Lee C.M., Kim J.H., Park S. (2022). Comparative risk of anemia and related micronutrient deficiencies after Roux-en-Y gastric bypass and sleeve gastrectomy in patients with obesity: An updated meta-analysis of randomized controlled trials. Obes. Rev..

[22] Nie Y., Tian Z., Wang P., Liu B., Zhang N., Zhou B., Wang S., Hei X., Meng H. (2023). Prevalence of anemia and related nutrient deficiencies after sleeve gastrectomy: A systematic review and meta-analysis. Obes. Rev..

[23] Riley R.D., Ensor J., Snell K.I.E., Harrell FEJr Martin G.P., Reitsma J.B., Moons K.G.M., Collins G., van Smeden M. (2020). Calculating the sample size required for developing a clinical prediction model. BMJ.

[24] McCracken E., Wood G.C., Prichard W., Bistrian B., Still C., Gerhard G., Rolston D., Benotti P. (2018). Severe anemia after Roux-en-Y gastric bypass: A cause for concern. Surg. Obes. Relat. Dis..

[25] Lee Y.C., Lee T.S., Lee W.J., Lin Y.C., Lee C.K., Liew P.L. (2012). Predictors of anemia after bariatric surgery using multivariate adaptive regression splines. Hepatogastroenterology.

[26] Ben-Porat T., Elazary R., Sherf-Dagan S., Weiss R., Levin G., Rottenstreich M., Sakran N., Rottenstreich A. (2020). Factors Associated with the Development of Anemia During Pregnancy After Sleeve Gastrectomy. Obes. Surg..

[27] Bailly L., Schiavo L., Sebastianelli L., Fabre R., Pradier C., Iannelli A. (2018). Anemia and Bariatric Surgery: Results of a National French Survey on Administrative Data of 306,298 Consecutive Patients Between 2008 and 2016. Obes. Surg..

[28] Wang T.Y., Huang H.H., Hsieh M.S., Chen C.Y. (2020). Risk of anemia in morbidly obese patients after bariatric surgery in Taiwan. World J. Diabetes.

[29] Chung J.O., Park S.Y., Cho D.H., Chung D.J., Chung M.Y. (2018). Anemia is inversely associated with serum C-peptide concentrations in individuals with type 2 diabetes. Medicine.

[30] González-Domínguez Á., Visiedo-García F.M., Domínguez-Riscart J., González-Domínguez R., Mateos R.M., Lechuga-Sancho A.M. (2020). Iron Metabolism in Obesity and Metabolic Syndrome. Int. J. Mol. Sci..

[31] Ikeda-Taniguchi M., Takahashi K., Shishido K., Honda H. (2022). Total iron binding capacity is a predictor for muscle loss in maintenance hemodialysis patients. Clin. Exp. Nephrol..

